# Species-specific analysis of protein sequence motifs using mutual information

**DOI:** 10.1186/1471-2105-6-164

**Published:** 2005-06-29

**Authors:** Jan Hummel, Nima Keshvari, Wolfram Weckwerth, Joachim Selbig

**Affiliations:** 1 Max Planck Institute of Molecular Plant Physiology, Am Mühlenberg 1, D-14424 Potsdam, Germany; 2 University of Potsdam, Institutes of Biochemistry/Biology and Computer Science, c/o Max Planck Institute of Molecular Plant Physiology, Am Mühlenberg 1, D-14424 Potsdam, Germany

## Abstract

**Background:**

Protein sequence motifs are by definition short fragments of conserved amino acids, often associated with a specific function. Accordingly protein sequence profiles derived from multiple sequence alignments provide an alternative description of functional motifs characterizing families of related sequences. Such profiles conveniently reflect functional necessities by pointing out proximity at conserved sequence positions as well as depicting distances at variable positions. Discovering significant conservation characteristics within the variable positions of profiles mirrors group-specific and, in particular, evolutionary features of the underlying sequences.

**Results:**

We describe the tool ***PRO****file analysis based on ****M****utual ****I****nformation *(PROMI) that enables comparative analysis of user-classified protein sequences. PROMI is implemented as a web service using Perl and R as well as other publicly available packages and tools on the server-side. On the client-side platform-independence is achieved by generally applied internet delivery standards. As one possible application analysis of the zinc finger C_2_H_2_-type protein domain is introduced to illustrate the functionality of the tool.

**Conclusion:**

The web service PROMI should assist researchers to detect evolutionary correlations in protein profiles of defined biological sequences. It is available at  where additional documentation can be found.

## Background

Here we describe PROMI, a system to discover group-specific conservation characteristics in the amino acid distribution of profiles. For this we understand the sequences forming a general profile to be associated with a user-defined biological classification label, where the number of labels should be much smaller than the number of rows in the profile. In detail relations between profile columns and the applied group affiliation of the sequences forming the profile shall be investigated. The relations will be apparent by constituting significant amino-acid conservations, leading either to distinct amino acid consensus patterns in the analyzed groups or to knowledge about affinity between the groups [1].

To tackle this aim the mutual information (MI) is used as an interdependence measure of random variables *X*_*i *_and *Y *[2-5]. The interdependence between *X*_*i *_(in our case column of a profile *X*) and *Y *(here group affiliation) is understood as the knowledge one gains about *Y *if *X*_*i *_is known and vice versa [6,7]. Small values imply small gain of knowledge between the variables, whereas high values point out a higher gain. The calculated MI-profile of the whole alignment consisting of all *k *groups as well as all  pairwise profiles together with computed sequence logos finally allow conclusions regarding group-specific amino acid-positions where the distribution differ significantly and thus a group-discrimination on the basis of one profile-position is possible. Moreover the mean value of each pairwise MI-profile leads to formation of an elementary distance matrix *D*, where low MI-profile-mean-values state that the molecular similarity between groups of sequences is high opposed to higher MI-profile-mean-values with a higher molecular distance in the underlying groups. Further, by applying hierarchical clustering to *D*, a phylogenetic tree reflecting the distance between its constituents can be constructed.

In the following we use "class" and "classification" synonymously with "group" and "group affiliation".

## Implementation

**Figure 5:**
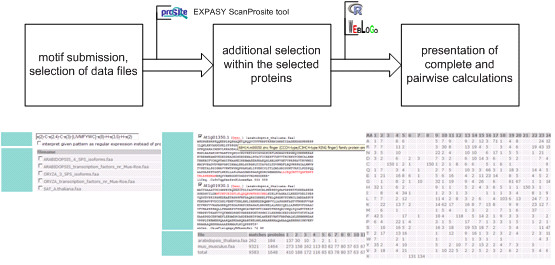
Workflow of the web service PROMI. In step one the user specifies the motif and selects (may be user submitted) protein files. For sane results step two can be used to refine the selection derived by step one (by disabling false positive matches) and to limit the matches for a balanced ratio within the classes. Step three is the presentation of the complete and pairwise calculations in plots (compare Figure 2) as well as tables breaking down the multiple alignments into ratios of amino acids per position.

PROMI is implemented in Perl as a web based service running on an apache web server and available for free use. Depicted in Figure 5 the selecting of matches relating to consensus sequences in PROSITE format [8] or given as a regular expression is performed by using the EXPASY ScanProsite tool [9], a Perl reference implementation for dealing with PROSITE motifs. The chosen instances of the motif were aligned using ScanProsite as well and the organism-specific origin was assigned by splitting up the NCBI none redundant protein database file [10] into species-specific "proteome" flatfiles. By upload of user-prepared sequences in FASTA format any other user-defined classification, alternative to the classification by organism identifier, can be applied. All calculations are implemented in the R environment [11]. To fulfil this, the RSPerl [12] and RSvgDevice [13] packages were used to embed R inside of Perl and to offer high order output in svg-format rather than the default png-format (svg output requires a plug-in for the web browser as provided by Adobe [14]). The computation of the sequence logos is done on the server-side by local utilisation of the Berkeley weblogo software. The Bioperl [15] module Bio::SeqIO is used to handle files of protein sequences.

## Results and discussion

**Figure 1:**
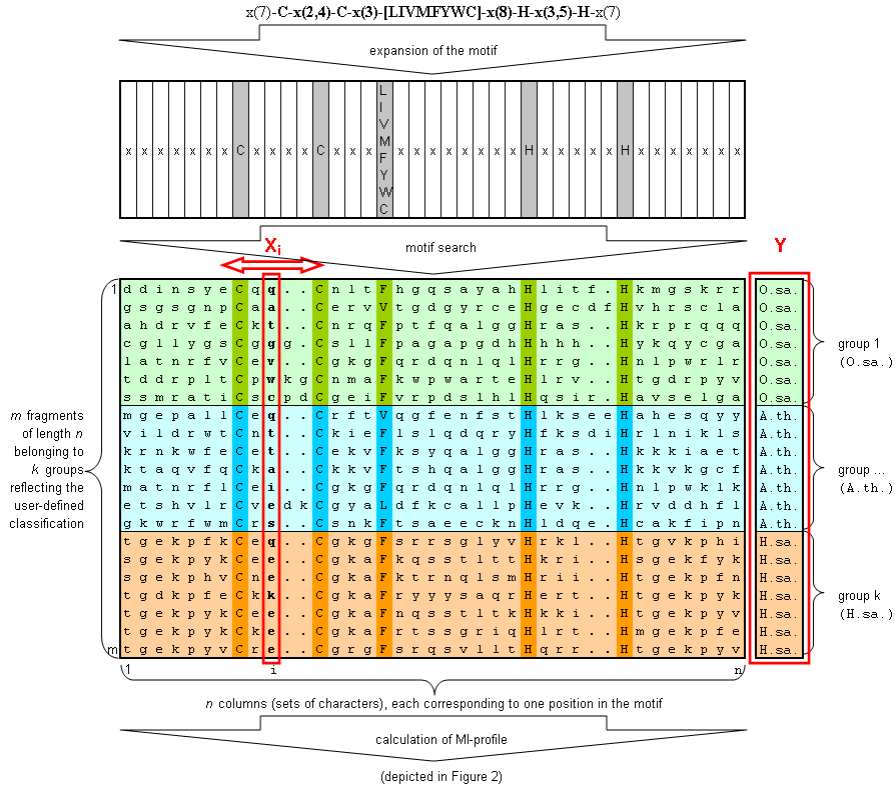
Schematic multiple alignment with *m *fragments of length *n *belonging to *k *user defined groups. During the MI-profile-calculation used vectors *X*_*i *_and *Y *are marked in red, whereas *X*_*i *_behaves like a sliding window from left to right (*i *= 1...*n*). *X *= (*X*_1_,..., *X*_*i*_,..., *X*_*n*_*) *and *Y *are derived by applying the enlarged Prosite motif PDOC00028 as a search-pattern to user-defined groups of protein sequences. By selecting fragments corresponding to the motiv a quasi multiple alignment was formed. In multitask the vector *Y *is formed from the user-given group labels. Here, to get a general idea, the alignment is colored and arranged per group-label of the vector *Y*. For demonstration conserved positions within the alignment (corresponding to the motif) are darkend. In case of pairwise alignments, only 2 groups would be stated.

Sliding a window from column *1 *to *n *of the profile, as can be seen in Figure 1, leads to a MI-profile for the motif where low MI-values correspond to positions with a high degree of conservation among their constituent groups, whereas high MI-values correspond to positions with good discriminatory power in terms of the applied group affiliation. For evaluation of significance a label permutation test giving a *p*-value for each mean and deviation is performed. Therefore the MI-profile resulting from the given (unshuffled) multiple alignment is compared against histograms of 100 profiles derived by shuffling each column of the underling multiple alignment. By observing separation of shuffled and unshuffeld values in both histograms (synonymous to a p-value near zero) a positive supporting factor for significance is given.

### Visualisation of mutual information profiles

**Figure 2:**
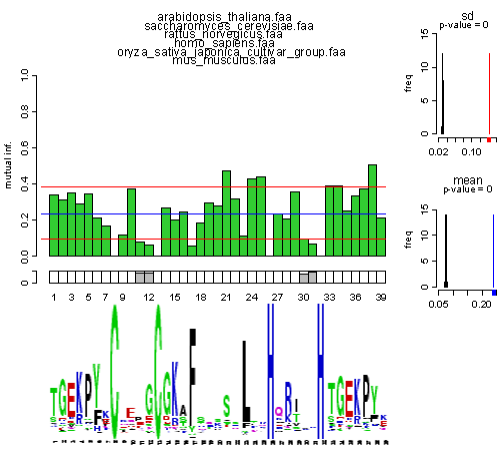
MI-profile for a multiple alignment of sequences derived from 6 organisms (top) and the corresponding Sequence logo (bottom). Each bar corresponds to one column in Figure 1, reflecting the value for the mutual information between the position *X*_*i *_and the a priori applied user-classification *Y *of the underlying multiple alignment.

Figure 2 shows a computed MI profile at the top and the corresponding sequence logo aligned below. The profile is derived by applying the PROSITE motif PDOC00028 with the consensus pattern C-x(2,4)-C-x(3)-[LIVMFYWC]-x(8)-H-x(3,5)-H for the Zinc finger C_2_H_2_-type domain on several organism-specific "proteomes" using the tool PROMI. To take the structurally important linker region in zinc finger proteins of animals into consideration this pattern was enlarged at the N-and C-terminus by 7 amino acids. Each bar within the MI-profile corresponds to a specific column of the multiple alignment showing the obtained MI-value. As can be seen the MI-values differ over the alignment and the cysteines and histidines at positions 8, 13, 26, and 32 show the lowest MI-values (0.0) since these amino acid positions have to be conserved among all sequences. The blue line indicates the mean level, and two red lines correspond to the standard deviation. The row under the bar plot indicates the gaps' frequency according to the alignment (white/grey ratio equals no gaps/gaps ratio). The histogram to the top right explicitly shows the MI standard deviation of 100 shuffled sample sets joined by the unshuffled standard deviation value marked in red whereas the histogram below (bottom right) shows the same for the mean (in blue). Thus, the histograms provide significance levels by showing the separation of shuffled and unshuffled MI-values. All MI-values larger or smaller than the marked red lines are therefore obvious significant positions with organism-specific differences within the amino acid distributions. At the bottom of Figure 2 the sequence logo derived by the Berkeley weblogo tool [16] is depicted. Sequence logos are computed using entropy, and thus reveal the predominant amino acids available at specific positions within multiple alignments. Strong conservation between species is illustrated particularly lucidly at positions 8, 13, 26, and 32, which show very low MI-values. In contrast high MI-values point out positions with a high discrimination between the species, as visualized for positions 21, 24 and 25. The increased MI-values in the region of positions 33–38 coincides with the well described animal-specific TGEKP motif [17]. However, each position within the motif is valued differently – especially position 38 – suggesting a more differentiated view on this motif in a species-specific comparison.

### Phylogenetic tree

**Figure 3:**
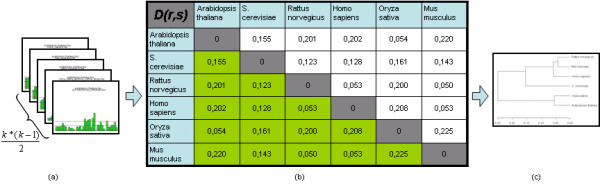
Construction of distance matrix *D(r,s) *(b) in our case of *k *= 6 groups by calculating each mean of all  pairwise MI-profiles (a). *r *and *s *are placeholder of the user-applied classification labels, where *D(r,s) *is the distance of a group of sequences labeled *r *to a group of sequences labeled *s *and visa verse. By applying hierarchical clustering with the complete linkage method to *D *a phylogenetic tree reflecting the distances can be constructed (c).

**Figure 4:**
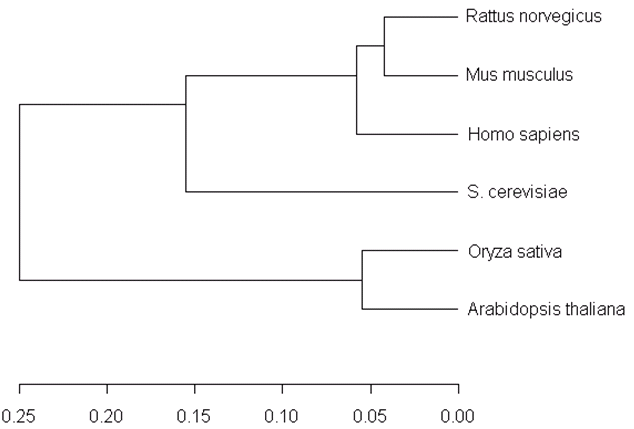
Phylogenetic tree derived by using the mean of the pairwise computed MI-profiles as distances. The scale provides the absolute distance values of the build clusters.

In case of *k *given groups (see Figure 1)  pairwise MI-profiles can be constructed. The resulting MI-average of each pair provides a distance matrix interpretable as a species-specific distance measure (schematically given in Figure 3), which is sensitive enough to reflect relations in the underlying profiles. As can be seen in Figure 4, the resulting phylogenetic tree for the C_2_H_2_-domain of comprised species *Arabidopsis thaliana*, *Homo sapiens*, *Mus musculus*, *Oryza sativa*, *Rattus norvegicus *and *Saccharomyces cerevisiae *reveals the known phylogenetic relationship of the zinc finger domains of the observed organisms on the basis of group-and motif-specific differences. Of particular interest is the fact that *S. cerevisiae *is clustered with animals rather than with plants.

Seemingly a very naive and rough approach disregarding protein steric structures as well as amino acid proximities the method is coherent. MI as a distance measure satisfies the four axioms of a metric: non-negativity, identity of indiscernibles, symmetry and triangle inequality [18].

## Conclusion

As aforementioned the method we have outlined may not only be applied in a species-specific context, but may also be understood in terms of a phylogenetic identifier, gene expression values or any other desired classification by the user. Influence of potential negative thresholds, as for example false positive matches using PROSITE motifs for sequence search, or a possible bias because of highly differing amounts of matching sequence fragments per group, could be decreased with little extra effort. For this the inclusion of a user-prepared multiple alignment, derived by BLAST and CLUSTAL is conveniently possible. The number of selected instances per group can be balanced by limiting the fragments to a suitable number. Regardless of the abovementioned issues, the proposed method is a fast and convenient approach for the motif-specific analysis of hundreds of sequences derived by homology of ortholog or paralog gene and protein domain families.

## Methods

### Multiple alignment and mutual information

Given a motif, describing sequence fragments of length *n*, the potential *m *matching sequence fragments with length *n *can be arranged as a multiple alignment as seen in Figure 1. Thereby each of the *n *columns corresponds to one position of the motif. Furthermore the *m *fragments belong to *k *groups reflecting the user-applied classification (*m *>> *k*). By setting up this structure *n m*-dimensional vectors *X*_*i *_(with *i *= 1...*n*) are constituted, each consisting of characters from the amino acid one letter code plus one additional letter for gaps. All *X*_*i *_combine as the matrix *X *= (*X*_1_, *X*_2_,..., *X*_*i*_,..., *X*_*n*_) reflecting the profile. Additionally an *m*-dimensional vector (*Y*) is formed from the classification consisting of a discrete set of group labels, where each label corresponds per row to the group affiliation of a sequence fragment in matrix *X *at the same row.

A convenient measure of correlation between two discrete random variables such as in our case *X*_*i *_and *Y *is the mutual information *I*(*X*_*i*_, *Y*) using the entropy and joint entropy respectively



and has been applied recently [1-5].

The entropy *H*(*X*_*i*_) is calculated using the number of occurrences of each character *x *within the vector *X*_*i *_of length *m*. The same is used for *H*(*Y*). The joint entropy *H*(*X*_*i*_, *Y*) is derived by concatenating the characters *x *and the group labels *y *and again calculating the likelihood of all possible combinations. Furthermore each MI value is multiplied by a factor giving the likelihood of gaps in each column. This heuristic approach was adapted from the C4.5 machine learning algorithm [19]. Iterating each column *i *(*i *= 1...*n*) of the profile and calculating *I*(*X*_*i*_, *Y*) a so-called mutual information profile (MI-profile) can be established, incorporating all MI-values.

### Distance matrix and phylogenetic tree

Given sequences affiliated to *k *groups,  pairwise orderless combinations of groups can be formed. By calculating MI-profiles for all pairwise combinations so called pairwise MI-profiles can be created. As one potential measure the (k*k)-dimensional distance matrix *D *is formed up by calculating the mean over all columns of each pairwise MI-profile, schematically shown in Figure 3. This mean describes the distance of amino acid conservation between the groups of sequences. In the case of our study these groups are species-derived, but can also be of any other classification order. Given *r *and *s *as one possible combination of group labels  can be calculated. (*X*,*Y*)_*r*, *s *_are all these sequence fragments belonging either to group *r *or to group *s*. Eventually, the phylogenetic tree reflecting the distances can be constructed by applying hierarchical clustering (complete linkage clustering) to *D*.

## Availability and requirements

• Project name: PROMI

• Project home page: 

• Operating system: server side Linux; client side platform independent

• Programming language: Perl, R

• Other requirements: EXPASY ScanProsite tool, R, Bioperl, RSPerl, RSvgDevice, Berkeley weblogo software

• License: GNU GPL

• Any restrictions to use by non-academics: no

The source code of the web service script and the R script are available upon request. Other used software components are available at the according sources.

## Authors' contributions

JH assembled and developed the software components, conceived the idea of interpreting the MI as distance measure and participated in writing and drafting the manuscript. NK provided protein sequences, computed the biological results and drafted the manuscript. WW conceived the biological application of analysing the zinc finger C_2_H_2_-type protein domain. Further he interpreted the results and participated in the design of the study and drafted the manuscript. JS contributed to the conception of this study, participated in its design and coordination and helped to draft the manuscript. All authors read and approved the final manuscript.
